# The Psychosocial Impact of Congenital Cytomegalovirus on Caregivers and Families: Lived Experiences and Review of the Literature

**DOI:** 10.3390/ijns9020030

**Published:** 2023-05-26

**Authors:** Michelle P. Zappas, Amanda Devereaux, Megan H. Pesch

**Affiliations:** 1 Department of Nursing, Suzanne Dworak-Peck School of Social Work, University of Southern California, Los Angeles, CA 90015, USA; 2 National CMV Foundation, Tampa, FL 33606, USA; 3 Division of Developmental and Behavioral Pediatrics, Department of Pediatrics, University of Michigan Medical School, Ann Arbor, MI 48109, USA

**Keywords:** caregiver, family, congenital cytomegalovirus, burden of disease, indirect costs, spillover effects

## Abstract

Caring for a child with congenital cytomegalovirus (cCMV) can be costly for families, not only in terms of out-of-pocket expenses, but also in terms of caregiver time, relationships, career trajectories, and mental health. These additional burdens are sometimes referred to as “spillover effects”. As parents of children with cCMV, we, the authors of this article, discuss the impact that cCMV has had on our families. While multiple studies have reported on the epidemiology, prevention, screening, diagnosis, and management of cCMV, there has been minimal research regarding the possible impact on the family unit. In this narrative review, we discuss the various areas of the lives of families and caregivers that may be impacted by raising a child with cCMV. Whether children are minimally or severely affected by the sequelae of cCMV, they and their families merit the progression of awareness of the virus and governmental policies to help end cCMV. As the existing cCMV-specific literature is limited, we correlate studies of other childhood disabilities and find the mutuality experienced by families affected by cCMV.

## 1. Introduction

Congenital cytomegalovirus (cCMV) infection is a common cause of sensorineural hearing loss (SNHL) and neurodevelopmental disabilities in childhood, affecting 0.5% and 1.4% of live births in high-income and low-to-middle-income countries, respectively [1]. Due to its often clinically inapparent presentation, infants born with cCMV often go undiagnosed, and miss opportunities for early intervention and management [2]. Newborn cCMV screening programs have become more common over the last decade, owing to advances in testing technology and public health awareness, resulting in earlier diagnosis and treatment for conditions such as hearing loss [3]. Yet, the cost-effectiveness of such screening programs has been the subject of much debate [4]. Several studies have reported a large economic cost of cCMV or favorable cost-effectiveness of newborn screening [5,6], however a recent review pointed out several limitations of these studies to date [4]. While all studies considered estimates of healthcare costs, few included estimates of the indirect costs to caregivers and families. In their review, Grosse et al. conclude that “almost nothing is known about the overall economic impact of cCMV on families and societies, including parental time use”, which complicates any such economic assessment [7].

Behind the population level healthcare cost estimates for cCMV are children, individuals, and families whose everyday lives are shaped, in some way, by the condition. Caring for a child with cCMV can be costly for families not only in terms of out-of-pocket expenses, but also in terms of caregiver time, relationships, career trajectories, and mental health, to name a few. In healthcare economics these are sometimes referred to as indirect costs (direct costs being the actual monetary costs) or “spillover effects” [8,9]. Studies examining the impact of having a child with cCMV on parents and families are few [10,11,12]. 

A better understanding of experiences of families specific to cCMV is an important step towards a more inclusive assessment of the cost-of-illness. Therefore, in this narrative review we highlight the areas of family and caregiver lives that may be impacted by having a child with cCMV. Recognizing the spectrum of cCMV-related clinical sequelae, which range from no sequelae (~70%) to having long-term disabilities (~20–30%), we are cautious to avoid a generalization of the possible impacts of cCMV on all families [13]. As the existing cCMV-specific literature is extremely limited, we also draw parallels between studies of other childhood illnesses and what commonalities may be experienced by some cCMV families. Lastly, we also draw on our collective experiences as clinician–mothers, having taken this journey with our own children, and later with our patients families.

### Lived Experiences—A Note on Bias, Positionality, Privilege, and Ableism

In every form of research, the views of authors are subject to bias from their positionality, their worldview, and their values and beliefs, which are, in turn, shaped by their religious faith, gender, education, employment, race, ethnicity, social status, (dis)abilities, etc. [14,15]. We, the authors, are all public health and healthcare professionals (a physician, a registered nurse, a nurse practitioner, and a regional public health official). We are also all mothers of children with cCMV, our professions ironically overlapping with our personal lives. We come to the issue of cCMV from a place of educational and social privilege. Unlike many families, our insider knowledge of the healthcare system and financial resources have allowed our children to access high-quality medical care and intervention, which is unfortunately not the case for all children. Yet, we also come to the issue of parenting a child with cCMV having struggled through pregnancy complications, hospitalizations, surgeries, countless therapy appointments, sleepless nights, early intervention, special educational services, guilt, grief, joy, and uncertainty. We recognize that our exact experiences cannot be generalized; however our positionality allows a unique point of view. We are both mothers and clinicians. We were trained in the medical model of disability (the view that a disability is a problem, an aberration from “normal” and something to be cured) [16], and are also raising children with disabilities who we celebrate for their diversity. It is important to note that while we do not view our children as a burden, we also feel that it is important to acknowledge the reality of cCMV and its potential impacts on families. Like many parents, we could not imagine our lives without our children exactly as they are. Listing the positive qualities of our children with cCMV and the richness they have brought to our hearts and families is beyond the scope (and word limit) of this submission, or even this entire journal. However, herein, we focus on some of the areas of struggle which we refer to as negative impacts, as is consistent with the literature [17,18]. 

## 2. Methods

As is standard with narrative reviews, it is not intended to systematically capture an entire literature, nor is it to merge study results into a quantifiable outcome [19,20]. Herein, the areas of family and caregiver lives that may be impacted by having a child with cCMV are reviewed in a narrative fashion. These domains include caregiver psychology, caregiver health, family resources and family finances. We acknowledge that these domains may not apply to all families, and that there are likely other ways that families are affected by having a child with cCMV. Furthermore, the ways in which families experience potential stressors are diverse—what may be experienced as emotionally or financially devastating to one family may not be to another [21,22,23]. Social and financial resources, relationships, values, as well as culture and prior life experiences shape the ways in which a family may experience having a child with cCMV. Utilizing individual perspectives and incorporating their citations is a powerful methodology that adds authenticity, depth, and richness to this narrative review. Giving a voice to families and caregivers of children affected by cCMV acknowledges an inclusive and complex understanding of the subject matter. This narrative is not meant to encompass the experiences of all families, nor is it meant to be a review of the condition itself, or the existing literature on the public health economic impacts of cCMV disease or newborn screening. Rather we review areas in which cCMV may impact the lives of families that are not often accounted for in the literature, but are none-the-less, in our opinions, substantial.

## 3. Results

### 3.1. Psychological Impacts on the Caregiver and Parent

While parents of children with any potentially disabling condition may experience a range of emotions and psychological distress, parents of children with cCMV may experience unique stressors related to their child’s past, present, and future. Prior work has found that parents of children with cCMV may experience a lack of sufficient social supports, isolation, and emotional strain of the “ups and downs” associated with the unpredictability of the disease [24]. This stress may not be just associated with a single time in the family’s life (e.g., the diagnosis), but for some families, such as our own, stressors from the past, present, and future exist. 

#### 3.1.1. Stress about the Past

Anecdotal evidence from blog posts, letters, and family stories describes a sense of guilt or self-blame experienced by parents of children with cCMV, particularly mothers [25,26,27]. This has also been true for the authors in our personal experiences. Congenital CMV is, in theory, a preventable disease [28,29]. The virus is passed through the maternal circulation to the growing fetus in utero [30]. Implicit in this pathophysiology is that the mother, herself, first had to acquire CMV before it could be passed to the fetus. It follows that had the mother avoided getting the virus in the first place, the fetus would have never been congenitally infected. As such, public health awareness campaigns highlight behavioral changes that pregnant women can do to prevent cCMV [28,31,32,33]. Pregnant women are encouraged to diligently practice hand hygiene after diaper changes, avoid sharing utensils or food with young children, and avoid kissing young children on the lips to prevent cCMV [34]. It is no wonder that many mothers harbor the feeling that they were in some way responsible or to blame for their child’s cCMV and resultant disabilities [10]. Research of other preventable conditions has identified persistent maternal self-blame and shame for their child’s congenital condition, even if they were unaware of the potential effects of their actions during pregnancy on fetal development [35,36]. While mothers of children with cCMV are not to blame for their child’s condition, mothers may still experience these feelings, which may originate from a perceived failure of upholding the cultural norm of being a “good mother” and protecting one’s offspring from harm [36,37].

A common sentiment among cCMV mothers is “if I had only known about cCMV, I could have prevented this” [38], however cCMV risk reduction education is not part of or has only recently become standard in prenatal care in several countries [39,40,41,42]. Families of children with cCMV may experience anger towards the healthcare community, who they may perceive as not properly educating them about the risks and prevention of cCMV during pregnancy [10]. By not being educated about cCMV, mothers may feel stripped of their autonomy in retrospect. One mother writes that mothers of children with cCMV “...may be angry that (they) were never warned of the dangers of CMV exposure while pregnant and angry that (they) were not allowed to make informed choices while pregnant to protect (their) unborn child” [43]. This perceived denial of prenatal cCMV education, is in fact, highlighted by the most recent position statement on CMV in pregnancy from the American College of Obstetrics and Gynecology which does not recommend prenatal cCMV behavior change counseling as women may find it “impractical and burdensome” to implement [44]. The notion of filtering health information given to patients, in particular those who have been historically disenfranchised by the healthcare system, is not only outdated but also paternalistic [45]. 

Parents of children with cCMV may also feel as if they have to direct their child’s evaluation and medical care. This is especially important as studies have shown that healthcare provider knowledge about cCMV diagnosis and management to be low [46,47,48,49]. The awareness of and knowledge about cCMV is lacking in providers from specialties critical to the care of these children, including primary care providers, pediatric hospitalists, therapists, otolaryngologists, and audiologists [46,47,48,50,51,52,53,54,55]. With limited guidelines from professional organizations on the management of cCMV [44,56], caregivers may feel unmoored in navigating the care of their children [10,12]. These feelings may compromise trust in providers, and amplify feelings of helplessness and parenting stress, which, in turn, may impact well-being. 

Many children born with CMV will never receive a cCMV diagnosis [2]. Among those who are diagnosed, many of them will be identified only after they go through what has been called a “diagnostic odyssey” [57,58]. Research on other conditions have indicated that a diagnosis is very valuable to families, and the (sometimes long) process of obtaining a diagnosis can be exhausting, stressful, and costly [59,60,61]. Some families of children with cCMV have reported that they only received a diagnosis because they “pushed and pushed” to find a cause for their child’s symptoms. Additionally, parents of children with a missed or late CMV diagnosis may feel resentful that their child was deprived of early intervention services, monitoring, and potential antiviral treatment [10,62].

#### 3.1.2. Present Day Stressors

The stress of caring for a child with a complex medical condition has been well documented. In a meta-analysis, Cousino et al. found that parenting stress among parents of children with chronic medical conditions was not consistently associated with illness duration or severity, but was associated with parental responsibility for treatment management [63]. In other words, it is not the severity of the child’s condition that is associated with increased caregiver stress, but rather the perceived responsibility for the care of their child. Children with cCMV have a wide spectrum of possible sequelae, from no apparent impacts to life-limiting conditions [13,29]. Caregiver stress may not be tied solely to the severity of a child’s sequelae from cCMV, but also to the resources and supports available and the necessity of the family’s involvement. For instance, even though a child with asymptomatic cCMV with isolated SNHL may be considered to be less affected by the virus than others, the caregivers may still experience increased levels of stress if the responsibilities associated with that condition rest on their shoulders. These may include obtaining insurance coverage for hearing aids or cochlear implants, keeping these devices in good working order and on the child’s head, overseeing and teaching the child and the family sign language, taking the child to therapy and specialist appointments, navigating the special education process and advocating for services and accommodations [64]. Parents of children with even one sequela of cCMV may still take on a variety of additional roles and tasks in relation to supporting their child’s additional needs or disability.

It is not surprising that parent distress has also been linked to perceived child impairment, in children with chronic medical conditions [65]. Specific to childhood hearing loss, parent–child quality of life has been found to be lower in children with more severe hearing loss in one study [66]. However, other research found that the severity of hearing loss was not associated with parents’ stress, but having a child with hearing loss and other disabilities [66,67]. In this same study, having a child with additional behavioral or emotional difficulties was related to poorer parent mental health. This may hold true for some families of children with cCMV who may be at greater risk of clinically significant behavior and neurodevelopmental disorders in addition to physical disabilities [68]. 

Parents of children with cCMV may also experience stigma. This may occur regardless of the severity of a child’s cCMV sequelae, or presence of visible disabilities. There is a robust literature on the stigma experienced by parents of children with multiple disabilities, or visible disabilities [36,69,70]. Parents may feel judged, unwelcome in certain places with their child, and have to field questions from strangers in public, for instance “what is wrong with her?” [71]. Not only can this be painful, but it can also lead to isolation and feelings of hopelessness. In a study of the experiences of parents of children with hearing loss (not specifically due to cCMV), many described feeling demoralized from negative social attitudes [64]. Another study examined the experiences of mothers of children with intellectual disabilities, which reported stigmatization and discrimination [72]. Mothers may also experience stigma related to having an infant with cCMV by healthcare professionals who are misinformed about the contagiousness of the virus, and place infants with cCMV under isolation procedures in the hospital. Anecdotally, families have shared that clinical providers have declined to care for their child with cCMV due to unfounded concerns of transmission. Family members, friends, therapists, and childcare providers may also attempt to keep a child with cCMV at arm’s length, effectively “othering” the child and parents.

#### 3.1.3. Anticipatory Grief and Stress 

Parents of children with cCMV have reported worries and stress about their child’s future. Some parents express having to constantly be vigilant for new-onset or worsening symptoms, such as hearing loss or new diagnoses that become more apparent with age [10]. A qualitative study by Vandrevala et al. acknowledged the emotional burden of the uncertainty parents face regarding long-term outcomes of their children with cCMV. One parent summed up their experience of mounting diagnoses as “dawning realizations that are then confirmed” and being left with “dramatic uncertainty” [10]. Other parents and caregivers of children with cCMV have reported worrying specifically about their child succumbing to sequelae of cCMV, such as Sudden Unexpected Death in epilepsy, or a severe respiratory infection [12]. These concerns are not unfounded. In addition to the studies showing the mortality risk in children with cerebral palsy (CP) and epilepsy [73,74], tributes to “CMV warriors” who have died in infancy and childhood are not infrequent on social media and advocacy organization websites [75]. One author shares her perspective that “A couple of times a week usually I think about what life might be like without my daughter, and I can hardly breathe. No one should have to think about their child dying every day or every few days. The fear is paralyzing”. Visualizing what life will be like if the child dies at an early age is something that weighs heavily on some parents of severely affected children. 

Like many parents of children who are medically complex, parents of children with cCMV may also worry about what will happen if they are outlived by their child. A study of parents of children with intellectual and developmental disabilities reported that most parents hoped to outlive their child, and anticipated a lower quality of life for their child if this did not happen. Parents in this study also reported feelings of despair about who would fill their parenting role after their death [76].

### 3.2. Caregiver Health

For some families, caring for a child with a condition such as cCMV may divert attention from activities to support their own health [77,78]. Korndewal et al. conducted surveys measuring daily function and quality of life measures in 133 parents of children with cCMV, and 274 parents of children without cCMV [11]. The study reported greater physical and concentration problems and lower mental quality of life in parents with a child with cCMV and long-term impairments than those without a child with cCMV [11]. Studies on the health of family caregivers of children with disabilities have repeatedly identified greater likelihoods of a variety of health conditions, including chronic back pain, migraine, obesity, and respiratory conditions. This may be due to the increased likelihood of also engaging in health risk behaviors, such as smoking, drinking alcohol, and fewer hours of sleep [79]. Based on work examining health outcomes among caregivers of children with developmental disabilities in general, caregivers of children with more than one condition affecting their development (e.g., CP and autism spectrum disorder (ASD)) were at highest risk of poorer health outcomes (pooled estimate of studies 1.36; CI 0.80, 3.36; PI—0.64, 3.36) as compared to parents of children with one condition (e.g., Down syndrome) or parents of typically developing children [80]. This may be true for many families of children with cCMV, who are at increased risk of several such conditions. 

Anecdotally, this is a common theme among cCMV parents, who also post about these struggles on social media—caregivers of children with more medically complex needs post about taking the “night shift” to monitor tube feeds, check blood glucose levels, or give medications, while also maintaining a day job and running a household [38]. The physical care of children with additional needs to support their mobility (lifting and transferring in and out of beds, bathtubs, wheelchairs, etc.) can also wear on a caregiver. In addition to families of children more severely affected by cCMV, we hypothesize that having a child with just a single known sequelae of cCMV may also impact a caregiver’s ability to care for their own health and that of others in the family. For example, while an infant with cCMV may appear to have no sequelae or even isolated hearing loss at birth, the potential still exists for hearing loss or developmental delays to emerge. The period of early childhood may be particularly stressful for families as they navigate hearing loss, delayed milestones, and therapies to support developmental trajectories. As a result, caregivers may not have the resources to engage in health promotion activities (cooking balanced meals, exercising, getting sufficient sleep) or seek out preventative or more acute medical care for themselves [81].

### 3.3. Impacts on Functional Capacity and Human Capital within the Family

The family systems theory, which has long been applied to families of children with chronic conditions, recognizes the mutual influence of each family member’s strengths and needs on the finite resources of the family [82]. Having a child with cCMV may shift the distribution of resources in some families, which may not be easily re-equilibrated.

#### 3.3.1. Family Resources for Other Children

To date, there has been no research on the impact of having a child with cCMV on siblings in the nuclear family. Looking to the literature around hearing loss and cochlear implant users [83], one study found that as parents devote more attention to support the needs of their child with cCMV, time and attention may be diverted away from siblings. Siblings of disabled or chronically ill children have been referred to as “glass children”, because they may become hidden within the family system [84], and their needs may go unmet [85]. It should be noted that less than 20% of children with cCMV develop lifelong disabilities, and less than 10% experience multiple conditions [86]. As such, comparisons between studies of siblings of children with chronic illnesses and those with cCMV should be drawn with caution. 

Findings about the long-term outcomes of siblings of children with chronic illness and disabilities in general are conflicting, with some studies finding an increased risk of adjustment disorders, behavioral and social problems, isolation, depression, and anxiety [21,87,88,89]. Alternatively, other studies have reported more positive findings including heightened resilience, compassion, and maturity in those with a disabled sibling [90]. Regardless, parents may have less time and financial resources to devote to siblings. Medical and therapy appointments for the child with cCMV may take precedent over dance or swimming lessons of the siblings, for example. As parents need to accompany children to such appointments, there may be a real difference in the time that siblings without cCMV are able to spend with their parents. This has been the case of all of the authors, who have shifted around their schedules in order to accompany their child with cCMV to appointments, facilitate transportation to special schools, practice therapy exercises, and provide in-home care for their children with cCMV. Our children without cCMV have expressed dismay about this imbalance in parental attention and quality time. 

Sibling relationships may also be atypical, while this is not always a negative, some children may experience disappointment if their sibling with cCMV cannot meet their expectations. For example, one author with a child with SNHL related to cCMV is countlessly told that her typical child does not like her sister and “wants to play with someone who can hear”. This can feel overwhelming for parents already dealing with the guilt, shame, and stress of managing caring for children with chronic illness.

#### 3.3.2. Impact on the Marital/Partnership Relationship

Individual parents may respond to a child’s diagnosis of cCMV differently; some may grieve, some may spring into action, some may experience denial. Discord in marriage or life-partner relationships can arise when partners’ coping strategies seem at odds with each other. For parents of newly diagnosed deaf or hard-of-hearing children, they can be faced with a myriad of decisions, potentially re-structured roles within the partnership and redistributed responsibility [91]. Some of these decisions, for instance those around communication modalities can yield strong opinions and opportunity for conflict. Anecdotally, among the community of parents of children with cCMV, this also seems to hold true. Reports of divorce, martial strain, and disagreements among partners not uncommon on social media pages for cCMV families. There are no studies about strains or stresses in co-parenting or marital relationships with children with cCMV to date. 

Time spent together without children may be difficult for some families to achieve, especially for parents who are taking their children to therapy and medical appointments. Many parents with disabled children have expressed difficulty obtaining respite support from caregiving. Barriers to finding respite care may be many, from obtaining funding to finding a reliable and available provider [92]. One Australian study of families of children with developmental disabilities (Down syndrome and ASD) found respite care to be associated with higher marital relationship quality and lower individual levels of parenting stress [93]. Two authors have experienced challenges with family caregivers (e.g., grandparents) who could provide care when the child was younger, but have more difficulty as the child ages, becomes physically larger, and the family caregiver ages. 

#### 3.3.3. Social Relationships 

Some parents of children with cCMV have reported stigmatization, and friendships with other adults dissolving after the birth of their child [94]. Having a child with cCMV can be life-altering, and parents may find they have less in common with previous friends. One author reported difficulties connecting with parents of typically developing children as “…many just don’t get it”. The author shared that “I don’t really like to meet new parents. I just have to explain so much to get to a place where they might understand me. It feels better to just stay connected with the friends who know what I have been through, or to connect with people who are dealing with similar experiences”. Parents of typical children may also make comments, many times well-intentioned, that parents of disabled children find insensitive [95]. For instance, it can be difficult for parents to hear “I’m sorry” when a person learns that their child is disabled, or “I don’t know how you do it” or “you’re amazing”, and other ableist language. We, as parents, are not sorry that our children exist, just as they are, and as such do not find apologies appropriate. We are also imperfect parents who are doing our best and attempting to learn from our mistakes just like everyone else. Telling parents that they are amazing only emphasizes the ableist notion that our child’s condition is a misfortune or a struggle and that only “super” parents would be able to manage it [95,96].

### 3.4. Family Financial Impacts

To date, there is little published about the economic impact on families caring for children with cCMV. Families caring for children with disabilities in general spend three times as much money caring for children than those without disabilities [97]. These costs include items or services that may not be covered by insurance, such as hearing aids, cochlear implants, therapy, special food, supplements, and equipment. One study examined the self-reported equipment needs and out of pocket expenditures for families caring for children with cerebral palsy (CP) [98]. The sample of items of equipment was vast—from specialized seating/tables, car travel, mobility and communication devices, splints, orthoses, to equipment activities for daily living such as eating and toileting, and toys or recreation opportunities specific to the child’s disability [98]. 

Even for items or homecare services covered by health insurance, there is often a lengthy denial and appeals process many families must go through to have items covered [99]. One author spent over USD 8000 last year alone on speech therapy which may be reimbursed by the family’s insurance eventually, however each claim takes months to process without guarantee of approval. This process creates a barrier to accessing equipment or services and can be emotionally and physically exhausting for parents [99]. Even families with children with isolated SNHL may have difficulty accessing hearing devices [100]. For some, this financial hardship is a barrier to accessing sound, which, in turn, may delay their linguistic, communicative, and social skills [101]. 

More indirect costs of cCMV for families include loss of employment and earnings related to sick leave or leaving their jobs. The number of health care visits in a six-month period for children with disabilities is equal to approximately 50 vs. 0.29 for patients without disabilities [97]. After the initial diagnosis of cCMV, in the first year of the infant there are typically several appointments with each subspecialist on the child’s medical team, in addition to routine primary care and therapy sessions (e.g., physical therapy or speech therapy). One mother of a 2.5-year-old daughter with cCMV was recently asked by her employer to count the number of visits her daughter had attended to date related to her cCMV—she counted 220 visits in total. 

Caregivers often spend not only money on copays for these appointments but also on transportation to attend appointments, care for other children while the parent is attending the appointment, and even lodging and food if the medical center is far from home. As mentioned above, the time devoted to arranging and attending medical appointments can also make it challenging to maintain full-time employment. For single parents or families with limited incomes this may not be an option. In fact, research has shown that families with a disabled child have increased odds of experiencing food insecurity [102].

For some parents, having a child with additional healthcare needs in general may mean the loss of not only income but career advancement and professional identity [94]. Within cis–heterosexual couples, studies have shown that mothers (as opposed to fathers) of children with additional healthcare needs in general (e.g., ASD, or a chronic medical condition) are more likely to experience negative employment changes [103,104]. Mothers or female caretakers are more likely to make these personal sacrifices, such as reducing work hours or leaving employment altogether, to devote more time and energy to caregiving. Pinto and Raz explored employment outcomes for parents caring for children with ASD and hearing loss over a five-year period [105]. As compared to mothers of typically developing children, mothers of children with ASD and/or hearing loss had a greater decrease in workplace participation in the first year after birth, and unlike those with typically developing children, this rate did not recover after five years [105]. As a result, the overall household income of these families was lower than their typically developing counterparts, even after adjusting for potential confounders [105]. Societal expectations, norms, and financial considerations may play a role in these decisions. For the authors, this has been the case—we have reduced our work hours, switched to roles/employment with more flexibility and remote work, and shifted to less ambitious career tracks.

## 4. Conclusions

Despite its high prevalence and potential for long term developmental disabilities, cCMV is infrequently diagnosed in utero or at birth. As such, many infants with cCMV go unidentified and important opportunities for early intervention are missed. While there have been multiple studies conducted on the economic cost of cCMV and the cost effectiveness of newborn screening programs, the literature regarding indirect costs to families affected by cCMV is lacking. Parents with children impacted by cCMV will have a wide range of experiences. For many parents of severely impacted children, having a child with cCMV can alter many domains of their lives. We believe that the impact of having a child with cCMV on the family should be included in the calculus of the net benefit of newborn cCMV screening, awareness, and prevention programs. Future studies describing and quantifying the diverse impacts of cCMV on families are urgently needed. 

## Data Availability

No new data were created or analyzed in this study. Data sharing is not applicable to this article.
